# Supplementary figures and images for: Biochar application on paddy and purple soils in southern China: soil carbon and biotic activity
Source: R Soc Open Sci. 2019 Jul 10;6(7):181499. doi: 10.1098/rsos.181499 (PMC6689583; doi:10.1098/rsos.181499)

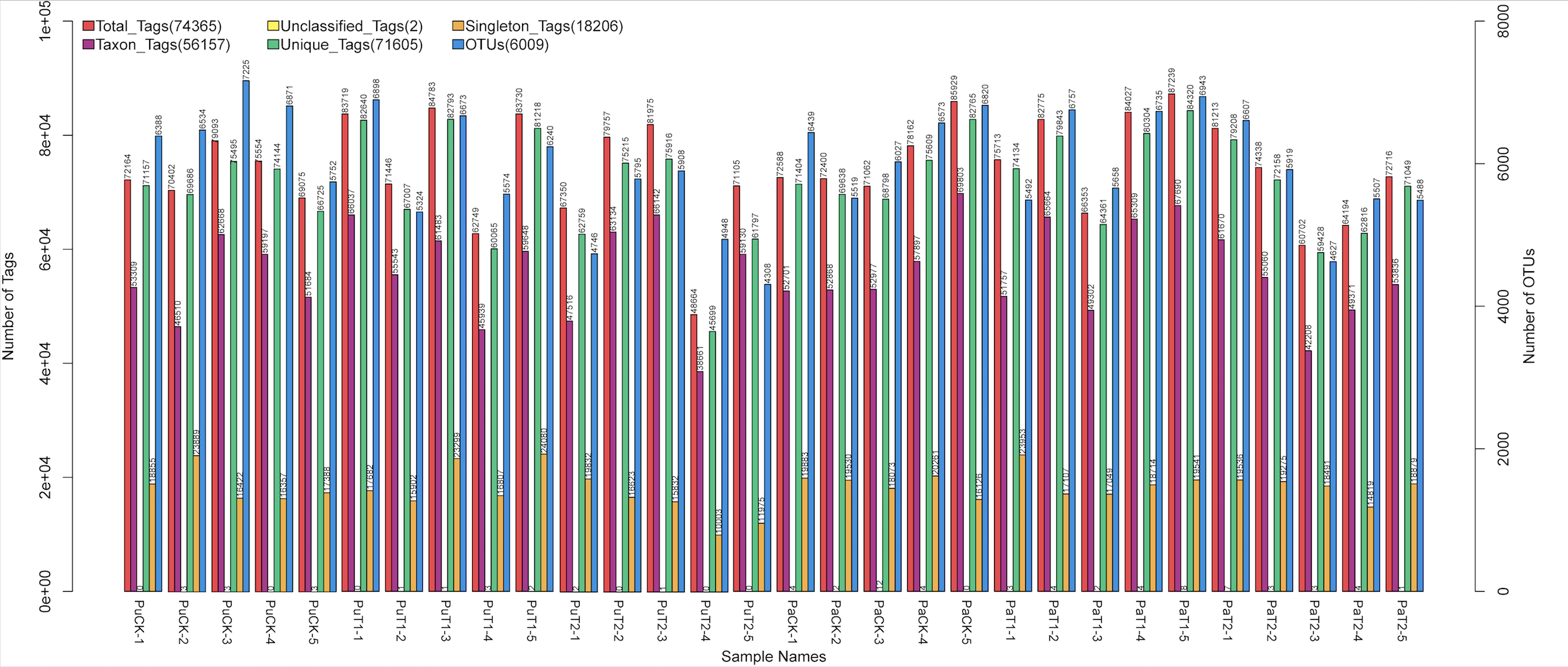

Supplement: Fig. S1 [file rsos181499supp1.png]

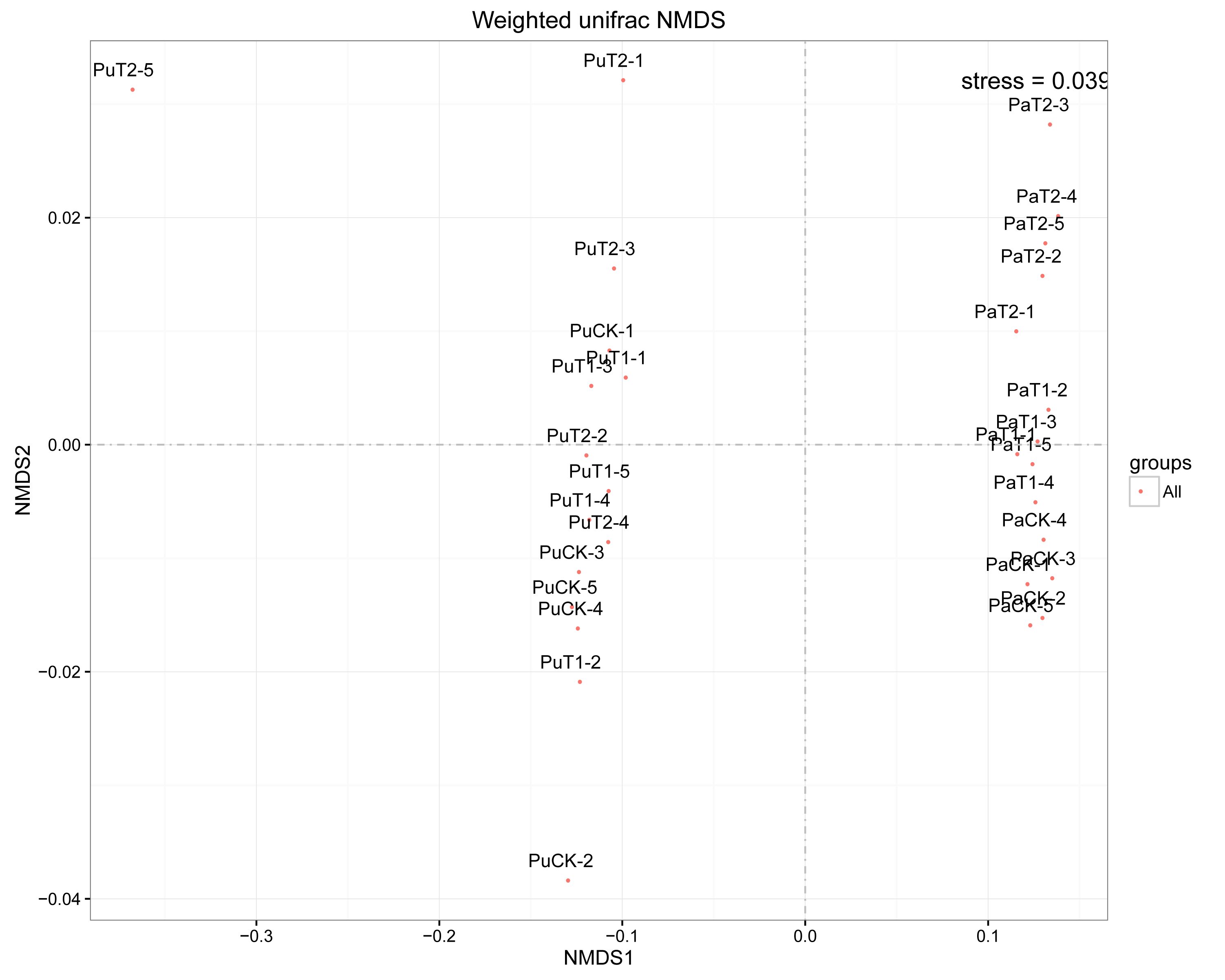

Supplement: Fig. S2 [file rsos181499supp2.tif]

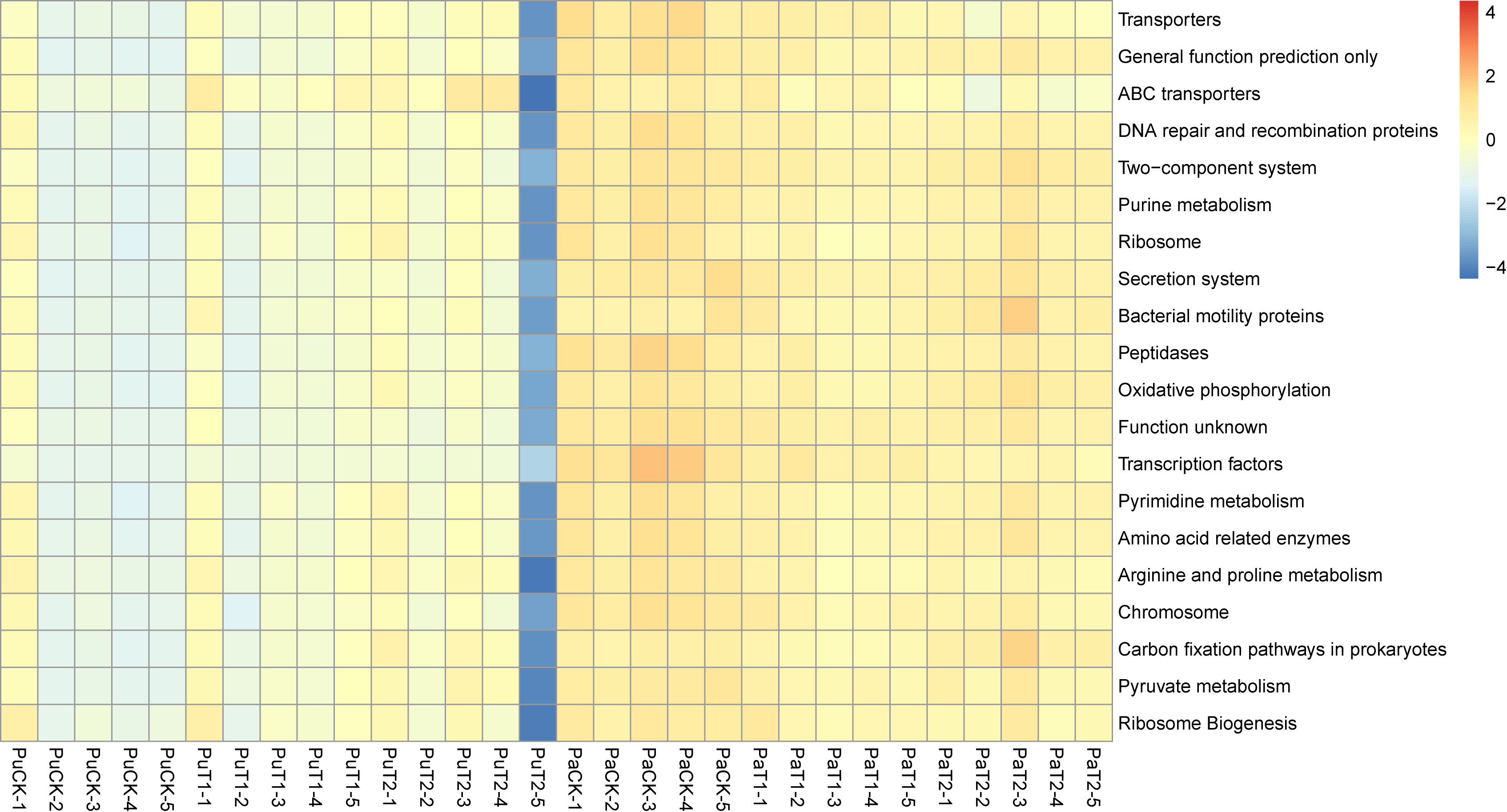

Supplement: Fig. S3 [file rsos181499supp3.tif]
